# Editorial Notes

**Published:** 1894-08

**Authors:** 


### Editorial Notes.

THE dengue fever has broken out at Key West, Fla.

THE *Doctor and Druggist* is the name of a new journal which has just made its appearance in St. Louis, Mo.

DR. JOSEPH M. MATHEWS was elected President of the State Board of Health of Kentucky at its last regular session.

DR. V. P. GIBNEY has been elected Professor of Clinical Surgery in the New York College of Physicians and Surgeons.

DR. R. HARVEY REED succeeds Dr. J. F. Baldwin as editor of the *Columbus Medical Journal.* Dr. Baldwin has occupied the editorial chair for eighteen years.

THE *Railway Surgeon* is a new medical monthly published in the interests of the railway surgeons. It appears from Chicago, and Dr. E. H. Reed is the editor.

THE St. Louis *Clinique* has passed into the hands of Dr. Emory Lanphear, Professor of Surgery in the College of Physicians and Surgeons. Dr. Lanphear will conduct the journal in the interests of that school and of the medical profession of the West.

THE ballroom of the Kimball House, Atlanta, Ga., has been secured for the next meeting of the Tri-State Medical Society of Alabama, Georgia and Tennessee, October 9th, 10th and 11th. The room is admirably adapted to the purpose, having fine acoustic properties and being entirely shut out from all noises from the street.

